# The Value of Ischemic Cardiac Biomarkers to Predict Spontaneous Breathing Trial or Extubation Failure: A Systematic Review

**DOI:** 10.3390/jcm13113242

**Published:** 2024-05-30

**Authors:** Carline N. L. Groenland, Maud A. Blijleven, Imane Ramzi, Eric A. Dubois, Leo Heunks, Henrik Endeman, Evert-Jan Wils, Vivan J. M. Baggen

**Affiliations:** 1 Department of Intensive Care, Erasmus MC, 3015 GD Rotterdam, The Netherlands; m.blijleven@erasmusmc.nl (M.A.B.); i.ramzi@erasmusmc.nl (I.R.); e.dubois@erasmusmc.nl (E.A.D.); leo.heunks@radboudumc.nl (L.H.); h.endeman@erasmusmc.nl (H.E.); v.baggen@erasmusmc.nl (V.J.M.B.); 2 Department of Cardiology, Thorax Center, Cardiovascular Institute, Erasmus MC, 3015 GD Rotterdam, The Netherlands; 3 Department of Intensive Care, Radboud University Medical Center, 6525 GA Nijmegen, The Netherlands; 4 Department of Intensive Care, Franciscus Gasthuis & Vlietland Ziekenhuis, 3045 PM Rotterdam, The Netherlands; e.wils@franciscus.nl

**Keywords:** ventilator weaning, ischemic cardiac biomarkers, SBT failure, extubation failure, intensive care

## Abstract

**Background:** It is unclear whether other cardiac biomarkers than NT-proBNP can be useful in the risk stratification of patients weaning from mechanical ventilation. The aim of this study is to summarize the role of ischemic cardiac biomarkers in predicting spontaneous breathing trial (SBT) or extubation failure. **Methods**: We systematically searched Embase, MEDLINE, Web of Science, and Cochrane Central for studies published before January 2024 that reported the association between ischemic cardiac biomarkers and SBT or extubation failure. Data were extracted using a standardized form and methodological assessment was performed using the QUIPS tool. **Results:** Seven observational studies investigating four ischemic cardiac biomarkers (Troponin-T, Troponin-I, CK-MB, Myoglobin) were included. One study reported a higher peak Troponin-I in patients with extubation failure compared to extubation success (50 ng/L [IQR, 20–215] versus 30 ng/L [IQR, 10–86], *p* = 0.01). A second study found that Troponin-I measured before the SBT was higher in patients with SBT failure in comparison to patients with SBT success (100 ± 80 ng/L versus 70 ± 130 ng/L, *p* = 0.03). A third study reported a higher CK-MB measured at the end of the SBT in patients with weaning failure (SBT or extubation failure) in comparison to weaning success (8.77 ± 20.5 ng/mL versus 1.52 ± 1.42 ng/mL, *p* = 0.047). Troponin-T and Myoglobin as well as Troponin-I and CK-MB measured at other time points were not found to be related to SBT or extubation failure. However, most studies were underpowered and with high risk of bias. **Conclusions**: The association with SBT or extubation failure is limited for Troponin-I and CK-MB and appears absent for Troponin-T and Myoglobin, but available studies are hampered by significant methodological drawbacks. To more definitively determine the role of ischemic cardiac biomarkers, future studies should prioritize larger sample sizes, including patients at risk of cardiac disease, using stringent SBTs and structured timing of laboratory measurements before and after SBT.

## 1. Introduction

The incidence and poorer outcomes of patients with weaning failure has been the subject of numerous studies over the past years [1,2]. Multiple studies have tried to identify factors related to weaning failure [3,4,5,6] in order to guide and optimize treatment of patients weaning from mechanical ventilation. Spontaneous breathing trials (SBTs) and the event of extubation are essential milestones during this weaning process. Although these steps can be regarded as different events, the underlying pathophysiological factors are broadly similar. Weaning failure is therefore often regarded as a composite of both SBT and extubation failure. SBT failure is defined as the inability to successfully tolerate spontaneous breathing with minimal support by PEEP and/or pressure support or via a T-piece for 30–120 min [7,8]. Extubation failure is defined as the need for reintubation within 48–72 h [9,10] or up to 7 days after extubation [11,12].

Despite significant efforts to improve the weaning process, extubation failure is still reported in up to 20% of patients [2,13]. Cardiac dysfunction has been shown to play an important role in both SBT and extubation failure [14,15]. Traditionally, the diagnosis of cardiac dysfunction during weaning failure involved invasive techniques such as Swan–Ganz measurements [16]. Studies have emphasized the need for better non-invasive predictors [17]. Cardiac biomarkers are promising candidates, as they are easy to perform, low-cost, and rapidly available. A previous systematic review indicated that despite successful SBTs, a rise in brain natriuretic peptide (BNP) was associated with extubation failure [4]. In addition, a rise in BNP levels was also observed in patients with SBT failure. High or increasing levels of BNP during weaning may be a reflection of either systolic and/or diastolic heart failure. Elevated levels of ischemic cardiac biomarkers (Troponin, CK-MB, Myoglobin) in critically ill patients have been documented during their ICU admission [18,19]. These markers can be increased during the early phase of the disease or during weaning from the ventilator (e.g., when reducing the level of inspiratory support and/or PEEP, or during an SBT [14,17]). Reducing the level of support and/or PEEP increases left ventricular pre- and afterload, and thus increases myocardial loading. The increase in myocardial oxygen demand can lead to an oxygen supply–demand mismatch that eventually results in myocardial ischemia and weaning-induced pulmonary edema [16,17,20,21]. Such an effect may be especially relevant in patients with (unknown) pre-existing heart failure or coronary artery disease. The potential value of ischemic cardiac biomarkers in predicting SBT and/or extubation failure is still unclear [17]. Therefore, the aim of this systematic review is to summarize the literature on ischemic cardiac biomarkers (Troponin-T, Troponin-I, Creatine Kinase-Myocardial Band (CK-MB), Myoglobin) and SBT or extubation failure.

## 2. Materials and Methods

The protocol of this systematic review was pre-registered in the PROSPERO international prospective register for systematic reviews (CRD 42023487261). The study was performed according to the Preferred Reporting Items for Systematic reviews (PRISMA) statement (Supplemental File S1). 

### 2.1. Data Sources and Search Strategy

The systematic search strategy as performed by the hospitals’ research librarian was carried out according to the PRISMA statement [22]. The following electronic databases were searched on 12 January 2024: Embase, MEDLINE, Web of Science Core Collection, Cochrane Central Register of Controlled Trials. Key search terms included: (1) weaning, extubation, and (2) ischemic cardiac biomarkers (Troponin-I, Troponin-T, CK-MB, Myoglobin). No language or publication date filters were applied. We searched for prospective and retrospective observational studies and randomized controlled trials (RCTs). A complete overview of the search strategy and the screened period for each database is provided separately (Supplemental File S2).

### 2.2. Study Selection Process

After deduplication, two authors (CG, MB) independently screened all initial search records on title and abstract. Subsequently, full-text screening was performed. A study was included if the following entry criteria were met: (1) Patient age ≥18 years, (2) Invasive mechanical ventilation >24 h in the Intensive Care Unit, (3) At least one ischemic cardiac biomarker reported, (4) SBT, extubation, or weaning failure/success reported. Disagreements were resolved in a consensus meeting, including the opinion of a third reviewer (VB). All references of the included articles were cross-checked to identify possible relevant articles missed in the original search syntax.

### 2.3. Study Endpoints

In this review, multiple endpoints were reported. We reported outcome on SBT failure, extubation failure, and weaning failure. Weaning failure was defined as a composite endpoint of SBT and extubation failure. 

### 2.4. Data Extraction and Quality Assessment

Relevant studies, baseline characteristics, biomarkers, and outcome data were extracted using a standardized form. Baseline characteristics of interest were age, sex, in- or exclusion criteria, method and duration of SBT (if performed). Data on timing of ischemic cardiac biomarker measurement were extracted (i.e., at ICU admission, any measurement during ICU admission, at day of extubation, before SBT, and/or after SBT). Finally, data on SBT, extubation, and weaning outcome (i.e., success or failure) were extracted. In this review, the values of Troponin-T and Troponin-I were reported in ng/L and those of Myoglobin and CK-MB were reported in ng/mL. If the included studies reported different units, the units were converted as appropriate. Both reviewers (CG and IR) independently performed a systematic methodological quality assessment of included studies using the QUIPS tool (Supplemental File S3). Disagreements were resolved in a consensus meeting, including the opinion of a third reviewer (VB). The potential risk of bias was rated as high, moderate, or low on six domains: study participation, study attrition, prognostic factor measurements, outcome measurements, study confounding, and ‘statistical analysis and reporting’.

## 3. Results

### 3.1. Search Results and Study Selection Process

**Figure 1:**
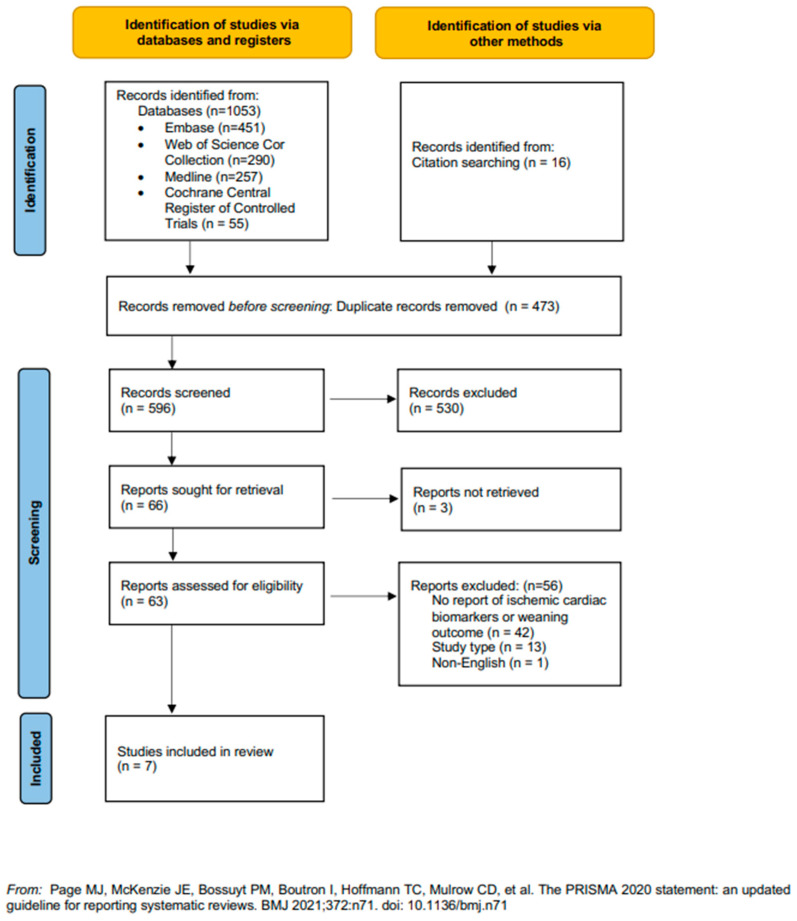
Flowchart of the study selection process according to the PRISMA statement [22].

The initial search strategy resulted in 580 records, and 16 records were retrieved by citation searching. After full-text evaluation of 66 potentially eligible records, 7 studies were included in the systematic review [23,24,25,26,27,28,29] (Figure 1). Study and patient characteristics of the included studies are presented in Table 1. The included studies were published between 2006 and 2021; they were either prospective (n = 5) or retrospective (n = 2) cohort studies. The study size ranged from 42 to 281 patients, mean age ranged from 51 to 69 years, and 53 to 64% of patients were male. In all studies, patients with underlying heart disease could be included. However, two studies excluded patients with limitations for routine echocardiography (e.g., non-SR, tachycardia, significant valvulopathy) [24,26], and one of those studies also excluded patients with a recent myocardial infarction or cerebrovascular accident [26]. The mean or median duration of mechanical ventilation before the first SBT ranged from 6 to 22 days. Five studies performed biomarker measurements before and after SBT [23,24,26,27,29], while one study performed measurements at ICU admission [28] and another reported the peak level during ICU admission [25]. 

### 3.2. Quality Assessment

**Figure 2:**
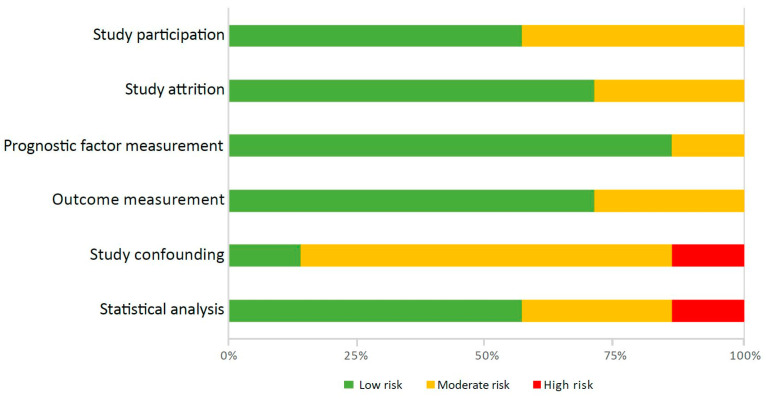
Summary of risk of bias of the included studies.

The methodological quality of the included studies is presented in Figure 2 and Supplemental File S4. In six studies, indications were present of moderate risk of bias in two [24,26,27] or three domains [23,25,29] per study. In the ‘Study confounding’ domain, five studies (71.4%) [23,25,26,27,29] had moderate risk of bias, and one study [24] had high risk of bias. Studies with moderate or high risk of bias did not adequately correct for confounders. In the domain of ‘Statistical analyses and reporting’, four studies had moderate risk of bias [23,26,28,29] and two studies had high risk of bias [24,27].

### 3.3. Study Endpoints

Among the included studies, different study endpoints were used, i.e., SBT failure, extubation failure, or weaning failure. The overview of the study endpoints is presented in Table 1. Two studies used SBT failure (defined according to predefined standardized criteria) as endpoint [23,29]. Three studies used extubation failure as study endpoint. Extubation failure was defined as either reintubation within 72 h [27,28] or reintubation at any point within the same hospital admission [25]. Two studies used weaning failure as study endpoint [24,26]. Weaning failure was defined as a composite of SBT and extubation failure within 48 h [26] or within the same hospital admission [24].

### 3.4. Ischemic Cardiac Biomarkers in Relation to Study Endpoints

All studies compared ischemic cardiac biomarkers at least at one single time point between success and failure patients [23,24,25,26,27,28,29]. In Table 2, all mean or median biomarker levels are presented, as reported by the studies.

Two studies found a relation between Troponin-I and the study endpoint [25,29]. Ionescu et al. found a higher peak cardiac Troponin-I in patients with extubation failure compared to patients with extubation success (50 ng/L [IQR, 20–215] versus 30 ng/L [IQR, 10–86], respectively, *p* = 0.01). Liu et al. found that Troponin-I measured before the SBT was higher in patients with SBT failure in comparison to patients with SBT success (100 ng/L ± 80 versus 70 ng/L ± 130, respectively; *p* = 0.03).

Konomi et al. reported a higher CK-MB measured at the end of the SBT in patients with weaning failure in comparison to patients with weaning success (8.77 ± 20.5 versus 1.52 µg/L ± 1.42, respectively; *p* = 0.047). Troponin-I and CK-MB measured at other time points were not found to be of predictive value. None of the studies reported a significant association of Troponin-T and Myoglobin with SBT or extubation failure.

### 3.5. Serially Assessed Ischemic Cardiac Biomarkers in Relation to Study Endpoints

Three studies additionally investigated the difference between serially assessed biomarker measurements before and after the SBT, stratified for the study endpoint. Bedet et al. measured Troponin-T before and after the SBT and did not observe a significant change during SBT in either success or failure group (*p* = 0.497, *p* = 0.546) [23]. Mottard et al. investigated the percentage increase in Troponin-T during the SBT and did not find a difference between patients with extubation success and failure (−2.4 ± 18.2 vs. −5.4 ± 11.6, *p* = 0.48, respectively) [27]. Lastly, Liu et al. measured Troponin-I before and 4 h after the start of the SBT and did not find a significant delta in patients with SBT success (*p* = 0.08) or SBT failure (*p* = 1.00) [29]. However, two studies [23,27] did not use paired statistical testing, which may have caused false negative study results.

## 4. Discussion

This is the first systematic review assessing the association between ischemic cardiac biomarkers (Troponin-T, Troponin-I, CK-MB, Myoglobin) and SBT or extubation failure. Among four ischemic cardiac biomarkers investigated in seven studies in this review, there is only limited evidence that Troponin-I and CK-MB are associated with SBT or extubation failure. One study found an association between Troponin-I (peak level during ICU admission) and extubation failure [25], while another reported an association between Troponin-I (measured before SBT) and SBT failure [29]. One study reported a higher CK-MB in patients with weaning failure measured at the end of the SBT [26]. All other studies investigating ischemic cardiac biomarkers did not find a significant association with weaning failure [23,24,27,28]. However, overall sample sizes were limited, and most studies had moderate to high risk of bias on confounding and statistical analysis.

We know from several meta-analyses that ischemic cardiac biomarkers are strongly related to mortality in the overall population of COVID-19 patients [30,31,32,33]. Moreover, specifically for COVID-19 patients admitted to the ICU, a prospective cohort study performed by Ghossein et al. showed an association between Troponin-T at admission and mortality [18]. Although this systematic review focused on extubation failure instead of mortality as study endpoint, we did expect some translation of these study results. We therefore found it surprising that most studies were not able to show a strong association between ischemic cardiac biomarkers and extubation failure. This raises the question whether relative myocardial ischemia due to supply–demand imbalance should be rejected as a contributing factor to weaning failure, or whether these studies were underpowered or biased to demonstrate such an association. Also, it is possible that in specific diseases such as COVID-19, other pathological mechanisms such as myocarditis explain the Troponin release. Of note, the work by Ionescu et al. [25] was the only study in this review that specifically included COVID-19 patients and did show a higher peak cardiac Troponin-I in patients with extubation failure compared to patients with extubation success.

The electrocardiogram (ECG) is regularly performed during the weaning process in daily clinical practice, and it is recommended as a diagnostic tool to diagnose stable coronary artery disease or myocardial ischemia [34,35]. Previous (older) studies have investigated the association between signs of ischemia based on the ECG and weaning failure, such as more than 1 mm ST segment deviation from baseline (ST depression or ST elevation) [36,37,38,39]. Evidence of ischemia based on the ECG was infrequently reported in the general population of patients weaning from mechanical ventilation; however, it tended to increase the risk of weaning failure [36] and to be associated with ventilator dependence [39]. Three studies included in this review [23,24,29] also reported on signs of ischemia based on the ECG. In these studies, ST-segment changes were variable among the study populations (6.8%, 12% and 23%). When they did occur, they were not accompanied by a change in troponin. However, ST-segment changes were more common in patients with prolonged weaning (13.7%) versus difficult (6.0%) and simple weaning (0%) [23]. Small sample sizes may have hampered the possibility to draw firm conclusions from these data. Considering the somewhat conflicting results in the literature, it could be worthwhile to further evaluate the role of the ECG in relation to ischemic cardiac biomarkers in larger and more recent studies.

Multiple reasons related to study design and methodological quality may explain why most of these studies were unable to demonstrate a significant association between ischemic cardiac biomarkers and SBT or extubation failure. First of all, it is unknown whether the half-time of the investigated cardiac ischemic biomarkers is short enough to demonstrate significant changes, for instance, when sampled directly after an SBT. For hs-Troponin, a cardiac stress test of 1 h should be enough to observe a significant rise [40]. Also, Myoglobin levels may be elevated in the serum within one hour after myocardial cell death [41]. However, for CK-MB, this interval might be too short. In the literature, a rise in CK-MB can be expected within 3–6 h after experiencing a myocardial infarction [42]. Nevertheless, these results are all based on the literature describing patients experiencing a myocardial infarction, and might not directly be translated to patients weaning from mechanical ventilation. This may explain why none of the studies were able to show an increase in ischemic cardiac biomarkers during the SBT in relation to SBT or extubation failure, although this was previously demonstrated for delta NT-pro BNP [4]. Secondly, the type and duration of SBT varied across studies (Table 1). Both might directly be related to cardiac biomarker release. Short and less challenging SBTs, i.e., 30 minutes and/or with CPAP, may lead to less cardiopulmonary stress and therefore lower biomarker release in comparison with a T-piece trial of 90 minutes [43]. Thirdly, like many studies in ICU patients, study populations were heterogeneous. Some studies did exclude specific patient categories, i.e., patients with a recent myocardial infarction or significant valvulopathy [24,26], while these patients could actually be most interesting for this research question. Furthermore, sample sizes were too small to perform subgroup analyses for patients with risk factors of myocardial ischemia (i.e., history of smoking; peripheral artery disease; previous cerebral vascular accident or myocardial infarction). However, in the study of Konomi et al., patients were stratified according to diastolic dysfunction [26]. In patients with diastolic dysfunction, Troponin-I together with BNP and Myoglobin were higher in comparison to patients without diastolic dysfunction. This is interesting because Bedet et al. showed that diastolic dysfunction has a role in weaning induced pulmonary edema, which is one of the major causes of weaning failure [23]. This suggests that ischemic cardiac biomarker measurements could be of value in specific patient populations.

Finally, the statistical analysis and reporting of the studies was overall moderate to poor. It is known that biomarker levels mostly have a skewed distribution; however, most studies did not describe the distribution of data. Almost all studies reported biomarkers as means with SDs, whereas skewed data should be reported as medians with interquartile ranges. This corresponds with the quality assessment of the studies were the domains ‘study confounding’ and ‘statistical analysis and reporting’ had high percentages of moderate and high risk of bias. In addition, four studies [24,26,27,29] reported sample sizes smaller than 100 patients. Apart from erroneously reporting mean values for skewed data in small groups, the reported reference values of Troponin-I (only reported in two studies) were also substantially different (normal < 400 ng/L [26] vs. normal < 1500 ng/L [24]). This may explain why the reported mean values especially for Troponin-I widely varied across studies. The presentation of data, the basic approach of statistical testing between (repeated) measurements, and the lack of study power may have caused false negative study results. Furthermore, only a few studies assessed CK-MB and Myoglobin, making it challenging to draw firm conclusions about these two biomarkers [24,26,28].

### 4.1. Study Limitations

We could not perform a meta-analysis due to the data presentation of the included studies, and therefore no combined results are reported. Furthermore, the definition of the study endpoint and the time point of biomarker measurements (at ICU admission/peak level/before SBT/after SBT) varied across studies. This limits the generalizability of the study results. Due to the large variation in the reporting of baseline characteristics of the included studies, we could not further elaborate on a potential association between clinical variables and biomarkers in patients with SBT/extubation failure or success. For example, blood pressure, saturation levels, and P/F ratios were only reported by three studies. Since lower blood pressure and saturation could contribute to cardiac ischemia, it would have been interesting to report the correlation between these clinical variables and ischemic cardiac biomarkers. This could further clarify which patients are at risk of failure and may even improve weaning strategies. Furthermore, some studies did not perform multivariable adjustment for factors such as the duration of mechanical ventilation, fluid balance before extubation, or comorbidities, all of which are known as predictors for extubation failure [3,5,44].

### 4.2. Clinical Implications and Future Perspectives

Based on the limited evidence that is currently available, ischemic cardiac biomarkers do not seem to have an important role in the prediction of SBT and extubation failure. We need future studies with larger sample sizes including patients at high risk of cardiac disease, and ideally sensitivity analyses should be performed in these patient groups. In addition, SBTs in patients at risk for cardiac disease must be stringent enough for the heart (which means zero PEEP and a reasonable duration). Moreover, biomarker measurements must be prospectively performed according to a structured protocol, i.e., before and after the SBT. In this systematic review, we only investigated the role of ischemic cardiac biomarkers; however, as previously shown for BNP [4], other cardiac biomarkers might have additional value as well. Over the past decade, there has been an increased interest in ‘novel’ cardiac markers (such as CA-125, ST-2, and Galectin-3). These markers are mostly related to risk stratification in heart failure [45,46,47,48]. However, more recent advancements have related, for example, CA-125 to respiratory failure in patients who were extubated after a successful SBT [49]. After multivariable adjustment, CA-125 levels exceeding 35 U/mL were associated with the occurrence of respiratory failure (OR 3.5, 95% CI 1.4–8.8). This association remained significant regardless of the presence of heart failure or higher lung ultrasound scores. In future studies, it would be of great interest to further investigate the value of these markers.

## 5. Conclusions

Considering the current literature, there is only limited evidence that Troponin-I and CK-MB are associated with SBT or extubation failure, and no evidence that Troponin-T and Myoglobin are associated with SBT or extubation failure. The lack of study power and methodological quality may have impeded the possibility to firmly demonstrate such an association. Future studies with larger sample sizes including patients at high risk of cardiac disease are needed to adequately address this hypothesis.

## Figures and Tables

**Table 1:** Main characteristics of the included studies.

Author, Year (Ref.)	Country	Study Design	Patients (n)	Study Inclusion Criteria	Duration of Mechanical Ventilation before 1st SBT (Days)	SBT Method and Duration	Biomarker	Time Points of Biomarker Measurements	Outcome
Frazier, 2006 [24]	Greece	Prospective cohort	43	MV > 48 h, sinus rhythm	12 ± 11	CPAP (5 PEEP), 120 min	Troponin-I, CK-MB	Baseline *, 1 and 24 h after start of the SBT	Weaning failure (composite of SBT or extubation failure within the same hospital admission)
Liu, 2016 [29]	France	Prospective cohort	81	Not specified	6 ± 7	T-piece, 60 min	Troponin-I	Before SBT and 4 h after SBT	SBT failure
Mottard, 2016 [27]	France	Prospective cohort	70	MV > 48 h, successful SBT	12 ± 8 **	Method unknown, 60 min	Troponin-T	120 min before SBT, 1 h after SBT	Extubation failure within 72 h
Konomi, 2016 [26]	Greece	Prospective cohort	42	MV > 48 h	22 ± 21	T-piece, 120 min	Troponin-I, CK-MB, Myoglobin	Before and at the end of the SBT	Weaning failure (composite of SBT or extubation failure within 48 h)
Bedet, 2019 [23]	France	Prospective cohort	208	MV > 48 h, patients who failed first SBT	6 [4–13]	T-piece, 120 min	Troponin-T	Before and at the SBT, 2 h after the start of SBT (only if the patient failed the SBT)	SBT failure
Yu, 2020 [28]	China	Retrospective cohort	125	MV > 24 h, no patients with pneumonia secondary to pulmonary diseases asthma/COPD	-	PS 5–7 cm H_2_O, 30 min	Troponin-T, Myoglobin	At ICU admission	Extubation failure within 72 h
Ionescu, 2021 [25]	USA	Retrospective cohort	281	MV, extubation attempt, positive SARS-CoV-2	-	-	Troponin-I	Peak level during ICU admission	Extubation failure at any point within the same hospital admission

MV = Mechanical ventilation, SBT = Spontaneous breathing trial, MI = Myocardial infarction, CVA = Cerebrovascular accident, CPAP = Continuous positive airway pressure, PS = Pressure support. * At study inclusion, unclear how long before the SBT. Duration of mechanical ventilation is presented in mean ± SD, or as median [IQR]. ** Duration of mechanical ventilation before extubation.

**Table 2:** Measurements of ischemic cardiac biomarkers per study endpoint (success/failure) measured before and after SBT.

Study/First Author	Success		Failure	
	Before SBT	After SBT	Before SBT	After SBT
**Troponin-T (ng/L)**				
Bedet [23]	47 [21–138]/297 ± 926	47 [21–165]/303 ± 969	54 [24–124]/410 ± 1866	53 [25–147]/418 ± 1921
Mottard [27]	214 ± 658	201 ± 647	110 ± 170	102 ± 159
Yu [28]	79 ± 187 ^†^	-	339 ± 1500 ^†^	-
**Troponin-I (ng/L)**				
Frazier [24]	690 ± 2460 ^††^	170 ± 360	110 ± 130 ^††^	70 ± 100
Liu [29]	70 ± 130 *	90 ± 60 ^†††^	100 ± 80 *	100 ± 60 ^†††^
Ionescu [25] ^††††^	30 [10–86] **	-	50 [20–215] **	-
Konomi [26]	60 ± 30	-	110 ±150	-
**CK-MB (ng/mL)**				
Frazier [24]	1.67 ± 2.05 ^††^	0.84 ± 1.02	2.61 ± 3.01 ^††^	0.63 ± 1.00
Konomi [26]	1.25 ± 0.73	1.52 ± 1.42 ***	8.78 ± 20.47	8.77 ± 20.5 ***
**Myoglobin (ng/mL)**				
Yu [28]	286 ± 558 ^†^	-	531 ± 869 ^†^	-
Konomi [26]	205 ± 171	-	279 ± 182	-

Values are presented as mean ± SD or as median [Interquartile Range] depending on presentation of the studies. Significant differences are indicated with an asterisk (*). ^†^ Measurement at admission to the ICU; ^††^ Measurement at study entry; ^†††^ Measurement at four hours after the start of the SBT ^††††^ Peak measurement during ICU admission; * *p*-value = 0.03, ** *p*-value = 0.011, *** *p*-value = 0.047.

## Data Availability

The datasets used during the current study are available from the corresponding author on reasonable request.

## Supplementary Materials

The following supporting information can be downloaded at: https://www.mdpi.com/article/10.3390/jcm13113242/s1. Supplemental File S1: PRISMA checklist; File S2; Search Strategy; File S3: QUIPS tool; File S4: Quality Assessment.
