# Supplementary material for: The Value of Ischemic Cardiac Biomarkers to Predict Spontaneous Breathing Trial or Extubation Failure: A Systematic Review
Source: J Clin Med. 2024 May 30;13(11):3242. doi: 10.3390/jcm13113242 (PMC11173145; doi:10.3390/jcm13113242)
Supplement: Supplementary file 1 [file jcm-13-03242-s001.zip › Supplemental File S2_SearchStrategy.pdf]

**Supplemental File S2 – Search strategy**

| Database searched | Platform | Years of coverage | Records | Records after duplicates removed |
|---|---|---|---|---|
| Embase | Embase.com | 1971 - Present | 451 | 444 |
| Medline ALL | Ovid | 1946 - Present | 257 | 44 |
| Web of Science Core Collection* | Web of Knowledge | 1975 - Present | 290 | 98 |
| Cochrane Central Register of Controlled Trials** | Wiley | 1992 - Present | 55 | 10 |
| **Total** | | | **1053** | **596** |

*Science Citation Index Expanded (1975-present) ; Social Sciences Citation Index (1975-present) ; Arts & Humanities Citation Index (1975-present) ; Conference Proceedings Citation Index- Science (1990-present) ; Conference Proceedings Citation Index- Social Science & Humanities (1990-present) ; Emerging Sources Citation Index (2005-present)

** Manually deleted abstracts from trial registries

**embase.com**

(((extubation/exp OR 'ventilator weaning'/de) AND (failure/de OR 'treatment failure'/de OR 'device failure'/de)) OR 'extubation failure'/de OR 'weaning failure'/de OR ((extubat* NEAR/6 (succes* OR fail*)) OR ((weaning OR liberat*) NEAR/10 (ventilat*) NEAR/10 (succes* OR fail*)) OR (weaning NEAR/6 (succes* OR fail*))):Ab,ti) AND ('biological marker'/de OR troponin/exp OR 'creatine kinase'/de OR myoglobin/exp OR (((cardiac OR biological*) NEAR/3 marker*) OR biomarker* OR bio-marker* OR ((blood* OR plasma*) NEAR/6 (level* OR concentration* OR analys* OR marker*)) OR troponin* OR creatine-kinase* OR myoglobin*):Ab,ti) NOT ([conference abstract]/lim AND [2000-2019]/py) NOT ([animals]/lim NOT [humans]/lim)

**Medline ALL Ovid**

(((Airway Extubation/ OR Ventilator Weaning/) AND (Treatment Failure/ OR Equipment Failure/)) OR ((extubat* ADJ6 (succes* OR fail*)) OR ((weaning OR liberat*) ADJ10 (ventilat*) ADJ10 (succes* OR fail*)) OR (weaning ADJ6 (succes* OR fail*))).ab,ti.) AND (Biomarkers/ OR Troponin/ OR Creatine Kinase/ OR Myoglobin/ OR (((cardiac OR biological*) ADJ3 marker*) OR biomarker* OR bio-marker* OR ((blood* OR plasma*) ADJ6 (level* OR concentration* OR analys* OR marker*)) OR troponin* OR creatine-kinase* OR myoglobin*).ab,ti.) NOT (exp animals/ NOT humans/)

**Web of Science**

TS=((((extubat* NEAR/5 (succes* OR fail*)) OR ((weaning OR liberat*) NEAR/10 (ventilat*) NEAR/10 (succes* OR fail*)) OR (weaning NEAR/5 (succes* OR fail*)))) AND ((((cardiac OR biological*) NEAR/2 marker*) OR biomarker* OR bio-marker* OR ((blood* OR plasma*) NEAR/5 (level* OR concentration* OR analys* OR marker*)) OR troponin* OR creatine-kinase* OR myoglobin*)))

**Cochrane CENTRAL**

(((extubat* NEAR/6 (succes* OR fail*)) OR ((weaning OR liberat*) NEAR/10 (ventilat*) NEAR/10 (succes* OR fail*)) OR (weaning NEAR/6 (succes* OR fail*))):Ab,ti) AND ((((cardiac OR biological*) NEAR/3 marker*) OR biomarker* OR bio-marker* OR ((blood* OR plasma*) NEAR/6 (level* OR concentration* OR analys* OR marker*)) OR troponin* OR creatine-kinase* OR myoglobin* ):Ab,ti)
