# Supplementary material for: The Value of Ischemic Cardiac Biomarkers to Predict Spontaneous Breathing Trial or Extubation Failure: A Systematic Review
Source: J Clin Med. 2024 May 30;13(11):3242. doi: 10.3390/jcm13113242 (PMC11173145; doi:10.3390/jcm13113242)
Supplement: Supplementary file 1 [file jcm-13-03242-s001.zip › Supplemental File S4_QualityAssessment.pdf]

Supplemental File S4 - Quality Assessment

	Study participation	Study attrition	Prognostic factor measurement	Outcome measurement	Study confounding	Statistical analysis
Frazier 2006	~	~	+	+	-	-
Liu 2016	+	+	+	~	~	~
Mottard 2016	~	+	+	+	~	-
Bedet 2019	+	~	+	+	~	~
Yu 2020	+	+	+	+	+	~
Ionescu 2021	+	+	~	~	~	+
Konomi 2022	~	+	+	+	~	~
